# Disturbance Estimation and Predefined-Time Control Approach to Formation of Multi-Spacecraft Systems

**DOI:** 10.3390/s24175671

**Published:** 2024-08-31

**Authors:** Zhicheng Zhang, Weimin Bao, Qimin Hou, Yinhao Ju, Yabin Gao

**Affiliations:** 1 College of Engineering, Peking University, Beijing 100871, China; zhicheng315@163.com; 2 China Academy of Aerospace Science and Innovation, Beijing 100871, China; 3 School of Aerospace Science and Technology, Xidian University, Xi’an 710071, China; 4 School of Astronautics, Harbin Institute of Technology, Harbin 150001, China; houqm@stu.hit.edu.cn (Q.H.); juyinhao@stu.hit.edu.cn (Y.J.)

**Keywords:** spacecraft formation, predefined-time control, sliding mode control, disturbance observer, fixed-time convergence

## Abstract

Accurate sensing and control are important for high-performance formation control of spacecraft systems. This paper presents a strategy of disturbance estimation and distributed predefined-time control for the formation of multi-spacecraft systems with uncertainties based on a disturbance observer. The process begins by formulating a kinematics model for the relative motion of spacecraft, with the formation’s communication topology represented by a directed graph for the formation system of the spacecraft. A disturbance observer is then developed to estimate the disturbances, and the estimation errors can be convergent in fixed time. Following this, a disturbance-estimation-based sliding mode control is proposed to guarantee the predefined-time convergence of the multi-spacecraft formation system, regardless of initial conditions. It allows each spacecraft to reach its desired position within a set time frame. The results of the analysis of the multi-spacecraft formation system are also provided. Finally, an example simulation of a five-spacecraft formation flying system is provided to demonstrate the presented formation control method.

## 1. Introduction

Nowadays, spacecraft formation technology has become a hot topic in the field of space research [1,2]. Spacecraft formation refers to a space system composed of two or more spacecraft that work together to accomplish a certain task while maintaining relative positions [3]. Spacecraft formation technology has broad application prospects in fields such as communication, navigation, and Earth observation, and is of great significance in improving the flexibility, reliability, and efficiency of spacecraft systems [4].

The control method of spacecraft formations is one of the key factors in achieving spacecraft formation tasks. Managing disturbances from large inertial components like thrusters and solar sails, along with the complex space environment, is essential for maintaining control precision. During flight, the control system must quickly and accurately maneuver the spacecraft for solar sail adjustments and orbital changes within limited time windows. With fuel constraints and maneuver time limits, optimizing for minimal energy use, shortest time, and maximum acceleration is critical. The control system must also suppress vibrations from flexible components like solar sails, and ensure robust, long-term operation with fault diagnosis and tolerance capabilities. By controlling the relative position and attitude between spacecraft, collaborative work and navigation can be achieved, effectively improving the overall performance of spacecraft systems. The spacecraft formation control method aims to maintain the relative position and set orbit between spacecraft, while considering constraints and communication delays to guarantee the steady operation of the formation spacecraft [5,6]. In recent years, significant progress has been made in the research of spacecraft formation control theories. In terms of finite time control approaches, researchers optimize control strategies to improve the response speed and accuracy of formation spacecraft, thereby enhancing the efficiency and reliability of the whole spacecraft system. For example, Liu et al. in [7] presented a fast terminal sliding mode control (SMC) to deal with distributed orbit synchronization control of spacecraft formation systems considering unknown external disturbances and communication time delays. The study in [8] designed a fuzzy-logic-system-based fast nonsingular terminal sliding mode control for a spacecraft system with inertial uncertainties, faults, and actuator saturations, and the robustness was improved. The authors in [9] presented an SMC in conjunction with adaptive estimation and a radial basis function neural network, to handle the problem of the finite-time attitude coordinated control of spacecraft formation flying systems with complicated disturbances and uncertainties. The study in [10] addressed the backstepping technique-based finite-time orbit control strategy of spacecraft formation systems with limited network communication. The work in [11] surveyed some machine learning approaches for spacecraft control design including the methods of the synthesis of controllers to stabilize the orbital or angular motion, the optimal interplanetary trajectories, and the formation control. The above literature review indicates that the finite-time control method for spacecraft formation is of great significance in improving spacecraft system performance, reducing energy consumption, and improving task execution efficiency. Further research will help promote the development of spacecraft formation technology and provide more possibilities and opportunities for future space applications.

In the process of multiple spacecraft formations or synchronization, various uncertainties and disturbances may be faced, including but not limited to orbital disturbances, communication delays, sensor noises, and environmental changes [12,13]. Orbital disturbances include the effects of Earth’s gravity, solar and lunar gravity, atmospheric drag, etc., on spacecraft orbits, which may lead to orbital deviations and instability, When communicating between spacecraft, there may be a risk of reduced response speed or loss of control of the formation control system due to signal transmission delays. For sensor noises, spacecraft in a formation usually need to obtain information such as position and velocity through sensors, and sensor noise and uncertainty may lead to increased control system errors. With the movement of magnitude radar, environmental conditions may change in the space environment, such as the impact of solar radiation, cosmic rays, etc., on spacecraft equipment and systems. Additionally, in a multi-spacecraft formation, there may be interactions or interference between spacecraft, such as electromagnetic interference, fuel-sharing effects, etc., which can pose challenges to formation control [14].

Some methods for handling disturbances in robust formation control include robust control, adaptive control, fuzzy control, disturbance-observed-based control, etc. [15,16,17]. These methods aim to enhance the robustness of the formation system to external disturbances, ensuring stable operation of the system in the face of disturbances and perturbations. As an important technical means in control systems, the disturbance observers aim to monitor and estimate internal and external disturbances in real-time, take corresponding measures to tackle the disturbances, and improve the robustness and stability of the system. Significant progress has been made in the research of disturbance observers in robust spacecraft formation control, providing an effective way to address various interferences encountered in spacecraft formation systems. For example, to enhance the performances of spacecraft formation, Jia et al. [18] designed control methods based on disturbance observers. By using an extended state observer for adaptive adjustment of disturbances in multi-agent systems, the control methods proposed in [19,20] improve the system’s robustness. In [21], Xie et al. studied the application of disturbance observers to external disturbances in spacecraft formation systems, achieving attitude stability of formation spacecraft. By using disturbance observers to handle uncertainty in spacecraft formations [22], the robustness of spacecraft formation systems has improved. Javaid et al. [23] explored the method of using disturbance observers for adaptive interference suppression in spacecraft formation control to guarantee the stability and performance of the formation system.

Building on the previous analysis, this paper presents a solution to the formation of multi-spacecraft systems (MSSs) in the presence of disturbances, utilizing a predefined-time sliding mode control approach [24,25] based on a disturbance observer. The main contributions of this study are summarized as follows:

(1) A model of a spacecraft formation flying (SFF) system is developed, formulating the multi-spacecraft dynamics model of relative motion while accounting for disturbances.

(2) A fixed-time disturbance observer is explored for the estimation of the disturbances without prior knowledge of them for spacecraft formation flying. Different from conventional finite-time disturbance observers, this observer ensures that the estimation errors of the disturbances converge to zero in a fixed time, independent of initial states.

(3) A strategy of composite control using a predefined-time stability method and SMC is proposed, integrating a feed-forward term from the fixed-time nonlinear disturbance observer. This allows each follower spacecraft to achieve desired positions within a predefined time frame determined by the design parameters alone.

The structure of this paper is as follows. Section 2 establishes the MSS model. Section 3 details the disturbance observer and predefined-time sliding mode controller, along with stability analysis. Section 4 provides numerical simulations to test the proposed controller. Section 5 concludes the paper.

The notions used in this paper are standard. In this study, for a vector $x={\left[x_{1},x_{2},\ldots ,x_{n}\right]}^{⊤}\in R^{n}$ and a constant α, ${\mathrm{sig}}^{\alpha }\left(x\right)={\left[|x_{1}{|}^{\alpha }\mathrm{sign}\left(x_{1}\right),|x_{2}{|}^{\alpha }\mathrm{sign}\left(x_{2}\right),\ldots ,|x_{n}{|}^{\alpha }\mathrm{sign}\left(x_{n}\right)\right]}^{⊤}$, where sign(·) is the signum function. |·| denotes the absolute value, while ${∥\cdot ∥}_{\infty }$ refers to the infinite norm of a vector or matrix. ⊗ stands for the Kronecker product.

## 2. System Description and Preliminaries

### 2.1. Definition of Reference Coordinate System

**Figure 1:**
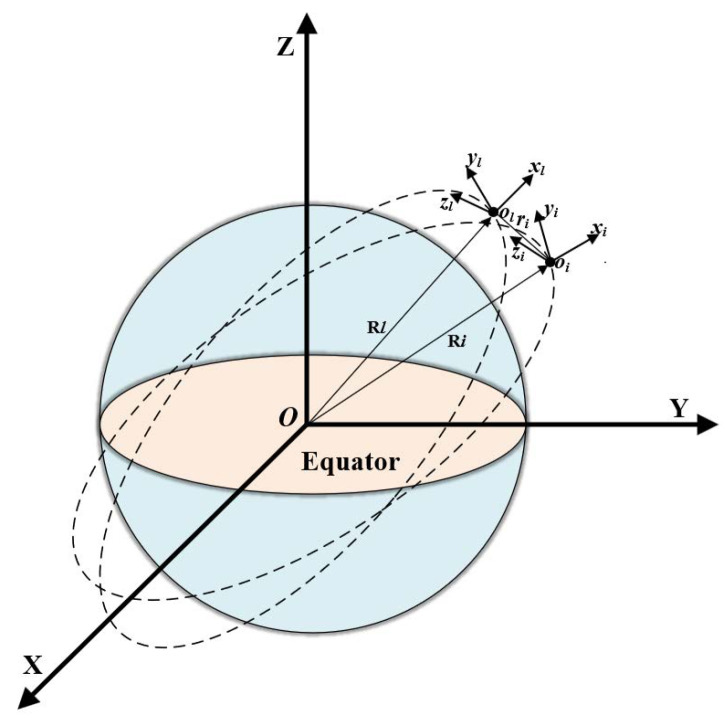
Reference spacecraft orbital coordinate system.

To begin with, it is essential to define the reference frames. F denotes the Earth-centered inertial frame, of which the origin *O* is the Earth’s center. In this frame, the X axis direction points to the vernal equinox; the Z axis points towards the North Pole; the Y axis lies in the Earth’s equatorial plane. Then, a right-handed coordinate system is formed. The frame $F_{l}$ represents the local vertical local horizon frame. Its origin $o_{l}$ is located in the (virtual) leader spacecraft’s center. In this frame, the direction of $R_{l}$ denotes the $x_{l}$ axis, the $y_{l}$ axis is aligned with the leader spacecraft’s orbital velocity direction, and the $z_{l}$ axis is fixed according to Cartesian coordinate rules. For the the *i*-th follower spacecraft of which the body-fixed frame is denoted by the frame $F_{i}$, its origin $o_{i}$ denotes the center of the *i*-th follower spacecraft; The principal coordinate axes are denoted by $x_{i}$, $y_{i}$, and $z_{i}$. Figure 1 shows these frames as described above.

For a formation with one virtual leader and n follower spacecraft, the translational dynamics of the *i*-th follower spacecraft is obtained in the LVLH frame as follows [26] Then, let us consider the relative position vector $r_{i}={\left[x_{i},y_{i},z_{i}\right]}^{⊤}=R_{l}-R_{i}$, where $R_{l}$ and $R_{i}$ represent the geocentric position vectors of the virtual leader and the *i*-th follower satellite/spacecraft, respectively. According to Newton’s law for universal gravitation, the second derivative of the relative position vector can be obtained within the equatorial inertial coordinate system F.

(1) $$\left\{\begin{array}{cc} \frac{d^{2}R_{l}}{dt^{2}} & =-\frac{\mu R_{l}}{R_{l}^{3}}+a_{l}\\ \frac{d^{2}R_{i}}{dt^{2}} & =-\frac{\mu R_{i}}{R_{i}^{3}}+a_{i} \end{array}\right.$$

The constant μ represents Earth’s gravitational parameter, specifically $\mu =3.9860047\times 10^{14}\;m^{3}/s^{2}$. Here, $a_{l}$ and $a_{i}$ denote accelerations resulting from external factors, primarily control forces and perturbations acting on the virtual leader and the *i*-th follower, respectively. The geocentric distances between the virtual leader and the *i*-th follower are denoted by $R_{l}$ and $R_{i}$, respectively. Next, we define the expression for the relative position vector $r_{i}$ to obtain its second-order derivative in the equatorial inertial coordinate system F [26]. From this, it follows that

$$\begin{array}{cc} \ddot{r_{i}}= & -2\omega \times \dot{r_{i}}-\omega \times \left(\omega \times r_{i}\right)-\dot{\omega }\times r_{i}\\ & -\mu \left(\frac{R_{i}}{R_{i}^{3}}-\frac{R_{l}}{R_{l}^{3}}\right)+\left(a_{i}-a_{l}\right), \end{array}$$

where ω stands for the instantaneous angular velocity vector of the virtual leader spacecraft orbit coordinate system $F_{l}$ relative to the equatorial inertial coordinate system F. Similarly, $\dot{\omega }$ represents the instantaneous angular acceleration vector of the virtual leader spacecraft orbit coordinate system $F_{l}$ over the equatorial inertial coordinate system F. For a formation comprising *n* follower spacecraft with one virtual leader, the follower spacecraft’s translational dynamics in $F_{l}$ are represented below:

(2) $$\begin{array}{cc} \ddot{r_{i}} & =A_{1}\dot{r_{i}}+A_{2}\left(r_{i}\right)+u_{i}+d_{i}, \end{array}$$

where

$$\begin{array}{ccc} A_{1} & = & \left[\begin{array}{ccc} 0 & 2\omega & 0\\ -2\omega & 0 & 0\\ 0 & 0 & 0 \end{array}\right]\\ A_{2}\left(r_{i}\right) & = & \left[\begin{array}{c} \dot{\omega }y_{i}+\omega^{2}x_{i}-\frac{\mu (R_{l}+x_{i})}{r_{i}^{3}}+\frac{\mu }{R_{l}^{2}}\\ -\dot{\omega }x_{i}+\omega^{2}y_{i}-\frac{\mu y_{i}}{r_{i}^{3}}\\ -\frac{\mu z_{i}}{r_{i}^{3}} \end{array}\right]\\ u_{i} & = & \left[\begin{array}{c} u_{xi}\\ u_{yi}\\ u_{zi} \end{array}\right]=\left[\begin{array}{c} a_{xi}\\ a_{yi}\\ a_{zi} \end{array}\right]-\left[\begin{array}{c} a_{xl}\\ a_{yl}\\ a_{zl} \end{array}\right], \end{array}$$

where $u_{i}$ and $d_{i}$ stand for the control vector and disturbances, respectively. For the external disturbance $d_{i}$, we assume that there exists unknown constants $d_{\max}>0$ and $d_{\max}^{*}>0$ that satisfy $∥d_{i}{∥}_{\infty }⩽d_{\max}$ and $∥{\dot{d}}_{i}{∥}_{\infty }⩽d_{\max}^{*}$.

### 2.2. Graph Theory

For the communication between the spacecraft, we use a graph to represent it. The communication for the spacecraft formation is considered as a leader–follower model which can be represented by a graph. G=(V,E) is a directed weighted graph of node *n*, in which V={1,2,…,n} represents a set of spacecraft nodes. E⊆V×V is a set of communication edges. It can be represented by ordered pairs of spacecraft. An edge (i,j) which is directed shows that the spacecraft *i* receives data from the spacecraft *j* but non-reversibly. If (j,i)∈E, then node *j* is called a neighbour node of node *i*. All neighbours of spacecraft *i* are denoted by $N_{i}=\left\{j|\left(j,i\right)\in E\right\}$. $A=\left[a_{ij}\right]\in R^{n\times n}$ describes an adjacency matrix, where $a_{ij}>0$ if (j,i)∈E, while $a_{ij}=0$ otherwise. $\Delta =\mathrm{diag}\{\delta_{1},\delta_{2},\ldots ,\delta_{n}\}$ denotes the degree diagonal matrix, where $D_{i}=\sum_{j=1}^{N}a_{ij}$ is the in-degree of spacecraft *i*. Thus, the Laplacian matrix of *G* is L=Δ−A. *G* contains a directed spanning tree if there is a spacecraft node (i.e., root node) that can guide all other spacecraft nodes through the directed path.

In this paper, we denote a graph $\bar{G}$ associated with *n* follower spacecraft and a virtual leader spacecraft. The virtual spacecraft is marked as 0 and the follower spacecraft are numbered by 1,2,⋯,n. In our scheme, we consider that the virtual leader spacecraft does not receive any data from the follower spacecraft, and only part of the followers can receive data from it. Thus, we denote $B=\mathrm{diag}\left\{b_{1},b_{2},\ldots ,b_{n}\right\}\in R^{n\times n}$, where $b_{i}$ is the weight of one-way communication from the virtual leader spacecraft to the *i*-th follower spacecraft. If the *i*-th follower spacecraft is connected to the leader spacecraft, then $b_{i}>0$; otherwise, $b_{i}=0$.

### 2.3. Definitions and Lemmas

Consider the following dynamic system:

(3) $$\dot{x}\left(t\right)=f\left(t,x\left(t\right)\right),\;t>t_{0},\;x\left(t_{0}\right)=x_{0},$$

where $f:R_{+}\times R^{n}\to R^{n}$ is a continuous function. For the system (3), assume that the origin is an equilibrium point. The following definitions and lemmas are introduced.

**Definition 1.**  *Consider the dynamical system *(*3*)*. The $T(x_{0})$, a function of $x_{0}$, is called the settling-time function of the system *(*3*)*, of which $x_{0}$ is the initial condition. Then, (i) the system is globally uniformly finite-time stable [27] if there exists a locally bounded function $T(x_{0})$: $R^{n}\to R_{+}\cup \left\{0\right\}$ such that $x(t,t_{0},x_{0})=0$ for all $t\geq t_{0}+T\left(x_{0}\right)$, where $x(t,t_{0},x_{0})$ is a solution of the system *(*3*)* with $x_{0}\in R^{n}$. The function T is called the settling-time function of the system *(*3*)*. (ii) The system *(*3*)* is fixed-time stable [28] if it is globally finite-time stable and there exists a constant $T_{\max}>0$ such that $T\left(x_{0}\right)\leq T_{\max},\forall \;x_{0}\in R^{n}$. (iii) The system *(*3*)* is predefined-time stable [29] if it is fixed-time stable and $T\left(x_{0}\right)\leq T_{p}$ for all $x_{0}\in R^{n}$ for any given parameter $T_{p}>0$.*

Below are some criteria of predefined-time stability and fixed-time stability, which will be used to the analysis of the formation and estimation errors.

**Lemma 1**  ([30]). *Consider the system *(*3*)*. The system *(*3*)* can be predefined-time stable if it holds that*

$$\dot{V}\leq -\frac{1}{T_{p}}\frac{2}{\alpha }\left(2V+V^{1-\frac{\alpha }{2}}+V^{1+\frac{\alpha }{2}}\right)$$

*where $T_{p}>0$ is any given parameter and α∈(0,1); $V\left(x\right):R^{n}\to R$ is a continuous positive definite function of the system states x.*

**Lemma 2**  ([30]). *Consider the system *(*3*)*. The system *(*3*)* can be predefined-time stable if it holds that*

$$\dot{V}\leq -\frac{1}{T_{p}}\frac{\pi }{\alpha }\left(V^{1-\frac{\alpha }{2}}+V^{1+\frac{\alpha }{2}}\right)$$

*where $T_{p}>0$ is any given parameter and α∈(0,1); $V\left(x\right):R^{n}\to R$ is a continuous positive definite function of the system states x.*

**Lemma 3**  ([31]). *Consider the system *(*3*)*. The system *(*3*)* can be globally fixed-time stable with its settling-time function $T(x_{0})$ satisfying*

$$T\left(x_{0}\right)\leq T_{f}≜\frac{1}{\alpha^{r}\left(1-pr\right)}+\frac{1}{\beta^{r}\left(qr-1\right)},\;\forall x_{0}\in R^{n},$$

*if it holds that (i) V(x)=0⇒x=0 and (ii) $\dot{V}\left(x\right)\leq -{\left(\alpha V^{p}\left(x\right)+\beta V^{q}\left(x\right)\right)}^{r}$, ∃α>0, β>0,p>0,q>0,r>0,pr<1,qr>1, where V(x) is a continuous positive function V(x) of the system states x.*

### 2.4. Problem Description

For the formation of the considered multi-spacecraft (2), let us define the formation errors ${\tilde{r}}_{i}$ and ${\dot{\tilde{r}}}_{i}$ of relative position and relative velocity to the virtual leader as follows: ${\tilde{r}}_{i}=r_{i}-r_{i}^{d}$ and ${\dot{\tilde{r}}}_{i}={\dot{r}}_{i}-{\dot{r}}_{i}^{d}$. In the the Earth-centered inertial frame, we consider that initial values $r_{i}\left(0\right)$ and ${\dot{r}}_{i}\left(0\right)$ are reasonable.

Thus, according to the definitions above, the desired formation of the MSS can be achieved in a predefined time if the ${\tilde{r}}_{i}$ and ${\dot{\tilde{r}}}_{i}$ are convergent in a predefined time $T_{f}\leq \max\left\{T\left({\tilde{r}}_{i}\right),T\left({\dot{\tilde{r}}}_{i}\right)\right\}$.

$$\begin{array}{cc} r_{i}-r_{i}^{d} & =0,\;\forall t\geq t_{0}+T\left({\tilde{r}}_{i}\right),\\ {\dot{r}}_{i}-{\dot{r}}_{i}^{d} & =0,\;\forall t\geq t_{0}+T\left({\dot{\tilde{r}}}_{i}\right),\;i=1,2,\ldots ,n, \end{array}$$

where $r_{i}^{d}$ represents the desired position of the *i*-th follower spacecraft, and ${\dot{r}}_{i}^{d}$ represents the desired velocity vector of the *i*-th follower spacecraft.

Hence, according to the definitions, we know that the convergence time of the formation errors ${\tilde{r}}_{i}$ and ${\dot{\tilde{r}}}_{i}$ can be flexibly adjusted according to the actual needs since the parameters $T_{c}$ and α can be predetermined.

To tackle the problem of MSS formation, this study explores a control scheme for a formation consisting of a single virtual leader and *n* follower spacecraft. The virtual leader is considered and designed to move in an optimal, undisturbed trajectory. The central goal is to devise a decentralized control strategy that includes a disturbance observer. This observer is designed to estimate the time-varying disturbances impacting the spacecraft formation. The strategy is intended to guarantee that the relative position and relative velocity of all follower spacecraft synchronize and precisely follow their desired states within a set time frame $T_{p}$. This synchronization is characterized by the convergence of $r_{i}$ to $r_{i}^{d}$ and ${\dot{r}}_{i}$ to ${\dot{r}}_{i}^{d}$ for each spacecraft, as time *t* approaches $T_{p}$.

## 3. Main Results

It is important to note that disturbances adversely affect trajectory tracking accuracy. To address this and enhance the robustness of the MSS, a fixed-time disturbance observer is developed to evaluate the disturbances for the MSSs. Following this, in order to achieve the desired formation within fixed time, by using the relative information and the virtual leader, a distributed predefined-time control law is designed based on advanced sliding mode techniques.

### 3.1. Fixed-Time Disturbance Estimation

Consider the second-order dynamics with the follower spacecraft (2). To formulate the fixed-time disturbance observer, recalling the formation errors ${\tilde{r}}_{i}=r_{i}-r_{i}^{d}$ (i=1,…,n) defined above, let estimation errors be defined as $\varepsilon_{1i}={\dot{\tilde{r}}}_{i}-{\hat{v}}_{i}$ (relative velocity estimation errors) and $\varepsilon_{2i}=d_{i}-{\hat{d}}_{i}$ (disturbance estimation errors) for the *i*-th follower spacecraft. Recalling the characters of the spacecraft system in (3), the following disturbance observer is formulated as

(4) $$\left\{\begin{array}{c} \begin{array}{cc} {\dot{\hat{v}}}_{i}= & -K_{1}{\mathrm{sig}}^{\alpha }\left(\varepsilon_{1i}\right)-K_{2}{\mathrm{sig}}^{\beta }\left(\varepsilon_{1i}\right)+{\hat{d}}_{i}\\ & +A_{1}\dot{r_{i}}+A_{2}\left(r_{i}\right)+u_{i}-{\ddot{r}}_{i}^{d}\\ {\dot{\hat{d}}}_{i}= & -K_{3}\mathrm{sign}\left(\varepsilon_{1i}\right) \end{array} \end{array}\right.$$

where ${\hat{v}}_{i}$ and ${\hat{d}}_{i}$ are used to estimate $d_{i}$. each $K_{i}\in R^{3\times 3}\left(i=1,2,3\right)$ is a diagonal matrix, in which the diagonal elements of $K_{3}$ satisfy $\lambda \left(K_{3}\right)\geq d_{\max}$. α and β are parameters which satisfy 0<α<1 and β>1.

**Theorem 1.**  *Consider the MSS with disturbance $d_{i}$ in *(*2*)* and the disturbance observer *(*4*)*. For some scalar 0<α<1 and β>1 and given positive diagonal matrices $K_{1},K_{2}$, and $K_{3}$ with $\lambda \left(K_{3}\right)\geq d_{\max}$, the disturbance observer can guarantee that the disturbance estimation error $\varepsilon_{2i}$ will converge to zero within a predetermined fixed time $T_{f}$, where the parameter $T_{f}$ is the upper bound of convergence time of the disturbance estimation errors.*

**Proof of Theorem 1.**  According to (2) and (4), the following dynamical system of the error system is derived:

(5) $$\left\{\begin{array}{c} \begin{array}{cc} {\dot{\varepsilon }}_{1i} & ={\dot{\hat{v}}}_{i}-{\ddot{\tilde{r}}}_{i}=-k_{1}\mathrm{sig}{\left(\varepsilon_{1i}\right)}^{\alpha }-k_{2}\mathrm{sig}{\left(\varepsilon_{1i}\right)}^{\beta }+\varepsilon_{2i}\\ {\dot{\varepsilon }}_{2i} & ={\dot{\hat{v}}}_{i}-{\dot{d}}_{i}=-k_{3}\cdot \mathrm{sign}\left(\varepsilon_{1i}\right)-{\dot{d}}_{i} \end{array} \end{array}\right.$$

Consider the candidate Lyapunov function

(6) $$V_{\varepsilon_{1i}}=\frac{1}{2}\varepsilon_{1i}^{⊤}\varepsilon_{1i}$$

The derivative of $V_{\varepsilon_{1i}}$ can be described as

$$\begin{array}{cc} {\dot{V}}_{\varepsilon_{1i}} & =\varepsilon_{1i}^{⊤}{\dot{\varepsilon }}_{1i}\\ \; & =\varepsilon_{1i}^{⊤}(-K_{1}{\mathrm{sig}}^{\alpha }\left(\varepsilon_{1i}\right)-K_{2}{\mathrm{sig}}^{\beta }\left(\varepsilon_{1i}\right)\\ & -K_{3}\int_{t_{0}}^{t}\mathrm{sign}\left(\varepsilon_{1i}\right)d\tau -d_{i})\\ \; & \leq -K_{1}{\left(\varepsilon_{1i}^{⊤}\varepsilon_{1i}\right)}^{\frac{\alpha +1}{2}}-K_{2}{\left(\varepsilon_{1i}^{⊤}\varepsilon_{1i}\right)}^{\frac{\beta +1}{2}}-K_{3}\left|\varepsilon_{1i}\right|\left(t-t_{0}\right)\\ & \leq -K_{1}{\left(2V_{\varepsilon_{1i}}\right)}^{\frac{\alpha +1}{2}}-K_{2}{\left(2V_{\varepsilon_{1i}}\right)}^{\frac{\beta +1}{2}}\\ & \leq -\lambda_{\min}\left(K_{1}\right)2^{\frac{\alpha +1}{2}}V_{\varepsilon_{1i}}^{\frac{\alpha +1}{2}}-\lambda_{\min}\left(K_{2}\right)2^{\frac{\beta +1}{2}}V_{\varepsilon_{1i}}^{\frac{\beta +1}{2}}. \end{array}$$

Hence, by applying Lemma 3, one can find that the real convergence time $T_{1}\left(\varepsilon_{1i}\right)$ of $\varepsilon_{1i}$ converging to zero is with a fixed bound $T_{f}$:

$$T_{1}\left(\varepsilon_{1i}\right)\leq T_{f}:=\frac{2^{\frac{1-\alpha }{2}}}{\lambda_{\min}\left(K_{1}\right)\left(1-\alpha \right)}+\frac{2^{\frac{1-\beta }{2}}}{\lambda_{\min}\left(K_{2}\right)\left(\beta -1\right)}$$

Thus, it follows that $V_{\varepsilon_{1i}}\equiv 0$ and ${\dot{V}}_{\varepsilon_{1i}}\equiv 0$ for all $t\geq T_{1}$. Meanwhile, considering the $\varepsilon_{2i}=0$ is multiplicatively dependent on $\varepsilon_{1i}$, one can also find that $\varepsilon_{2i}=0$ converging to zero is with a fixed bound $T_{f}$. This completes the proof. □

**Remark 1.**  *In this study, the disturbance observer *(*4*)* is used to estimate the disturbances in fixed time. The convergence of the error is independent of control input $u_{i}$, but $u_{i}$ must ensure the system’s convergence.*

**Remark 2.**  *In this article, it is worth noting that the fixed-time disturbance observer is used rather than a “predefined-time” disturbance observer to estimate the disturbances. This choice is made because the former already performs adequately and ensures the convergence within a predefined time, provided that the time $T_{1}$ is less than the predefined time $T_{f}$, where $T_{f}$ is a pre-specified time and is independent of initial states of the system.*

### 3.2. Predefined-Time Control Design

In this study, a directed topology is used to represent formation communication nodes of the MSSs. Let us define an auxiliary error:

(7) $$\begin{array}{c} e_{i}=b_{i}{\tilde{r}}_{i}+\sum_{j=1}^{n}a_{ij}\left({\tilde{r}}_{i}-{\tilde{r}}_{j}\right), \end{array}$$

where $a_{ij}$ and $b_{i}$ are the entries of adjacency matrices *A* and *B*, respectively. Thus, the auxiliary error (7) is

(8) $$\begin{array}{c} {\dot{e}}_{i}=\left(b_{i}+\sum_{j=1}^{n}a_{ij}\right){\dot{\tilde{r}}}_{i}-\sum_{j=1}^{n}a_{ij}{\dot{\tilde{r}}}_{j}-b_{i}{\dot{r}}_{0} \end{array}$$

To achieved the predefined-time formation of the MSSs interested, we explore a predefined-time SMC profit from due to the strong robustness and fast response of SMC. Then, auxiliary sliding mode variable is designed as follows:

(9) $$s_{i}=\dot{e_{i}}+h\left(e_{i}\right),$$

where the function $h(e_{i})$ is designed to ensure the finite-time reachability of the sliding mode.

In this work, $h(e_{i})$ is selected as

(10) $$h\left(e_{i}\right)=\frac{2}{\alpha T_{p}}(2+V_{e_{i}}^{-\frac{\alpha }{2}}+V_{e_{i}}^{\frac{\alpha }{2}})e_{i},$$

where $V_{e_{i}}=\frac{1}{2}e_{i}^{⊤}e_{i}$. $T_{p}>0$ and 0<α<1 are given scalars. Obviously, from the equation in (9), one knows that $s_{i}=0$ when ${\dot{e}}_{i}=-h\left(e_{i}\right)$. Then, considering that

(11) $$\begin{array}{cc} {\dot{V}}_{e_{i}} & =e_{i}^{⊤}{\dot{e}}_{i}\\ \; & =-e_{i}^{⊤}e_{i}\\ & =-\frac{2}{\alpha_{1}T_{p}}\left(2V_{e_{i}}+V_{e_{i}}^{1-\frac{\alpha_{1}}{2}}+V_{e_{i}}^{1+\frac{\alpha_{1}}{2}}\right), \end{array}$$

we have $e_{i}\to 0$ as $t\to T_{p}$, according to Lemma 1 and (11). That is to say that $s_{i}=0$ can be reached in the predefined time $T_{p}$ independent to the initial condition e(0), which is a strong finite-time reachability.

Then, based on the designed sliding variable $s_{i}$ and the sliding mode $s_{i}=0$, we derived the following distributed control law based on SMC with disturbance estimates ${\hat{d}}_{i}$ for the formation of the MSS:

(12) $$\begin{array}{ccc} u_{i} & = & u_{ri}-{\hat{d}}_{i}+(\sum_{j=1}^{n}a_{ij}+b_{i}{)}^{-1}\\ & & \times \left[\begin{array}{c} u_{si}+\sum_{j=1}^{n}a_{ij}\left(u_{j}+{\hat{d}}_{j}-u_{rj}\right) \end{array}\right] \end{array}$$

where $V_{s_{i}}=\frac{1}{2}s_{i}^{⊤}s_{i}$ and

$$\begin{array}{ccc} u_{ri} & = & -\left(A_{1}\dot{r_{i}}+A_{2}\left(r_{i}\right)\right)+{\ddot{r}}_{i}^{d}\\ u_{si} & = & -\frac{2}{\alpha T_{p}}(2+V_{s_{i}}^{-\frac{\alpha }{2}}+V_{s_{i}}^{\frac{\alpha }{2}})s_{i}-\dot{h}\left(e_{i}\right)-\delta \mathrm{sign}\left(s_{i}\right) \end{array}$$

in which $T_{p}>0$, 0<α<1, and δ>0 are some given parameters.

When using the distributed control law, it is required in practice that the directed graph of the communication for the spacecraft formation has a directed spanning tree with the leader as the root spacecraft node. The following theorem states that the proposed control in (12) can guarantee the convergence of the formation errors.

**Theorem 2.**  *Consider the auxiliary error system with disturbances $d_{i}$ in *(*8*)* and control in *(*12*)*. For some given parameters $T_{p}>0$, 0<α<1, and δ>0, the errors in *(*8*)* will converge to zero within a predefined time $T_{p}$, where $T_{p}$ is the upper bound of the error converging. As a consequence, the formation errors ${\tilde{r}}_{i}$ and ${\dot{\tilde{r}}}_{i}$ will converge to zero within a predefined time $T_{p}$.*

**Proof of Theorem 2.**  According to the graph theory, the knowledge of Kronecker products, and the entire formation presented in Section 1, the control law (12) is reformulated:

$$U=U_{r}-\hat{d}+\left[{\left(\Delta +B\right)}^{-1}⊗I_{3}\right]\left[U_{s}+\left(A⊗I_{3}\right)\left(U+\hat{d}-U_{r}\right)\right]$$

which can be further represented as

$$\left[\left(\Delta +B\right)⊗I_{3}\right]\left(U+\hat{d}-U_{r}\right)=U_{2}+U_{s}+\left(A⊗I_{3}\right)\left(U+\hat{d}-U_{r}\right).$$

where

$$\begin{array}{cc} U & ={\left[u_{1}^{⊤},u_{2}^{⊤},\ldots ,u_{n}^{⊤}\right]}^{⊤},\\ U_{r} & ={\left[u_{r1}^{⊤},u_{r2}^{⊤},\ldots ,u_{rn}^{⊤}\right]}^{⊤},\\ U_{s} & ={\left[u_{s_{1}}^{⊤},u_{s_{2}}^{⊤},\ldots ,u_{s_{n}}^{⊤}\right]}^{⊤},\\ \hat{d} & ={\left[{\hat{d}}_{1}^{⊤},{\hat{d}}_{2}^{⊤},\ldots ,{\hat{d}}_{n}^{⊤}\right]}^{⊤}. \end{array}$$

Since Δ−A=L, we have

(13) $$U=U_{r}-\hat{d}+\left[{\left(L+B\right)}^{-1}⊗I_{3}\right]\left(U_{2}+U_{s}\right).$$

Define $e={\left[e_{1}^{⊤},e_{2}^{⊤},\ldots ,e_{n}^{⊤}\right]}^{⊤}$. According to (13), we obtain $e=\left[\left(L+B\right)⊗I_{3}\right]\tilde{r}$, from which the second derivative $\ddot{e}$ is

(14) $$\ddot{e}=U_{s}+\left[\left(L+B\right)⊗I_{3}\right]\tilde{d}.$$

Then, considering the derivative of $s_{i}$, i.e., ${\dot{s}}_{i}={\ddot{e}}_{i}+\dot{h}\left(e_{i}\right)$, we choose the Lyapunov function $V_{s}=\frac{1}{2}S^{⊤}S$, where $S={\left[s_{1}^{⊤},\ldots ,s_{n}^{⊤}\right]}^{⊤}$. The derivative of $V_{s}$ is derived as $\dot{V_{s}}=S^{⊤}\dot{S}.$ Substituting (13) into (14) yields

(15) $$\begin{array}{ccc} {\dot{V}}_{s} & = & S^{⊤}\left[\ddot{e}+\dot{h}\left(e\right)\right]\\ & = & S^{⊤}\left[-\frac{2}{\alpha T_{p}}\left(2+V_{s}^{-\frac{\alpha }{2}}+V_{s}^{\frac{\alpha_{2}}{2}}\right)S-\left(\delta ⊗I_{3}\right)\mathrm{sign}\left(S\right)\right]\\ & & +S^{⊤}\left[\left(L+B\right)⊗I_{3}\right]\tilde{d}\\ & = & -\frac{2}{\alpha T_{p}}\left(2V_{s}+V_{s}^{1-\frac{\alpha }{2}}+V_{s}^{1+\frac{\alpha }{2}}\right)-\delta ∥S∥\\ & & +S^{⊤}\left[\left(L+B\right)⊗I_{3}\right]\tilde{d}\\ & \leq & -\frac{2}{\alpha T_{p}}\left(2V_{s}+V_{s}^{1-\frac{\alpha }{2}}+V_{s}^{1+\frac{\alpha }{2}}\right) \end{array}$$

According to Lemma 1 and the results in (6), for some given parameters $T_{p}>0$ and 0<α<1, it can be concluded that S as well as e will converge to the zero in the predefined time $T_{p}$. Consequently, the formation errors can converge to zero within the predefined time. This completes the proof. □

**Remark 3.**  *The distributed control law *(*12*)* proposed in this paper is partly inspired by the approach in [32]. It is important to note that the control law in [32] relies on neighbors’ control inputs, increasing communication complexity. Furthermore, this approach in [32] may result in communication loops when there are cycles in the communication graph. In contrast, the proposed distributed SMC law *(*12*)* in this paper only requires information from one-hop neighbors, ensuring a fully distributed approach with minimal communication interaction. Additionally, this control law considers uncertain dynamics and disturbances, making it more applicable to practical scenarios.*

## 4. Numerical Simulations

**Figure 2:**
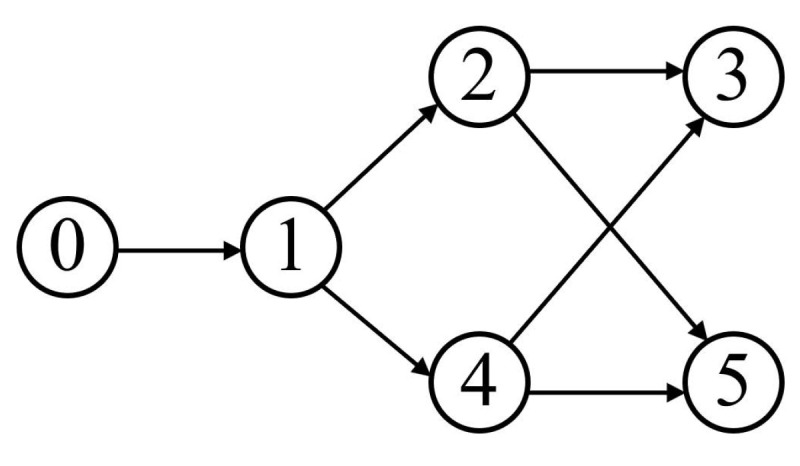
Communication topology.

In this section, a simulation example is provided to examine a scenario of formation of the MSS involving one virtual leader spacecraft and five follower spacecraft. Figure 2 illustrates the MSS’s communication topology.

In this topology, the weighted adjacency element $b_{i}$ is set to 1 for the case of a direct link from the virtual leader to the spacecraft *i*. Consequently, *A* and *B* are

$$A=\left[\begin{array}{ccccc} 0 & 0 & 0 & 0 & 0\\ 1 & 0 & 0 & 0 & 0\\ 0 & 1 & 0 & 0 & 0\\ 1 & 0 & 1 & 0 & 0\\ 0 & 1 & 0 & 1 & 0 \end{array}\right],\;\;B=\mathrm{diag}\left\{1,0,0,0,0\right\}.$$

Then, the Laplacian matrix and the degree matrix are

$$\begin{array}{c} L=\left[\begin{array}{ccccc} 0 & 0 & 0 & 0 & 0\\ -1 & 1 & 0 & 0 & 0\\ 0 & -1 & 1 & 0 & 0\\ -1 & 0 & -1 & 2 & 0\\ 0 & -1 & 0 & -1 & 2 \end{array}\right],\;\Delta =\mathrm{diag}\left\{0,1,1,2,2\right\}. \end{array}$$

Table 1 shows the parameters for the MSSs in simulations. The desired spacecraft formation information is as follows $r_{1}^{d}={\left[162,-235,280\right]}^{⊤}m$, $r_{2}^{d}={\left[-62,-380,-107\right]}^{⊤}\;m$, $r_{3}^{d}={\left[-200,0,-346\right]}^{⊤}\;m$, $r_{4}^{d}=\;{\left[-62,380,-107\right]}^{⊤}\;m$, $r_{5}^{d}={\left[162,235,280\right]}^{⊤}\;m$, ${\dot{r}}_{i}^{d}={\left[0,0,0\right]}^{⊤}\;m$, i=1,2,…,5.

Moreover, in simulations, the initial conditions of the spacecraft formation are as follows:

$$\begin{array}{cc} r_{1}\left(0\right) & ={\left[300,0,520\right]}^{⊤}\;m,\;{\dot{r}}_{1}\left(0\right)={\left[0.1,0.2,0\right]}^{⊤}\;m/s,\\ r_{2}\left(0\right) & ={\left[93,-571,161\right]}^{⊤}\;m,\;{\dot{r}}_{2}\left(0\right)={\left[-0.1,0,0.2\right]}^{⊤}\;m/s,\\ r_{3}\left(0\right) & ={\left[-243,-353,-420\right]}^{⊤}\;m,\;{\dot{r}}_{3}\left(0\right)={\left[-0.1,0.2,0.1\right]}^{⊤}\;m/s,\\ r_{4}\left(0\right) & ={\left[-243,353,-420\right]}^{⊤}\;m,\;{\dot{r}}_{4}\left(0\right)={\left[-0.2,-0.2,0.1\right]}^{⊤}\;m/s,\\ r_{5}\left(0\right) & ={\left[93,571,161\right]}^{⊤}\;m,\;{\dot{r}}_{5}\left(0\right)={\left[0.1,0,0.1\right]}^{⊤}\;m/s \end{array}$$

The disturbances used in simulations are represented in the following (i=1,2,…,5):

$$\left\{\begin{array}{c} d_{ix}=0.2\sin\left(\omega t+\frac{\pi }{3}i\right)\;{m/s}^{2}\\ d_{iy}=0.2\sin\left(3\omega t+\frac{\pi }{6}i\right)\;{m/s}^{2}\\ d_{iz}=0.2\sin\left(2\omega t+\pi i\right)\;{m/s}^{2} \end{array}\right.$$

The parameters of the predefined-time controller and fixed-time disturbance observer are shown in Table 2.

**Figure 3:**
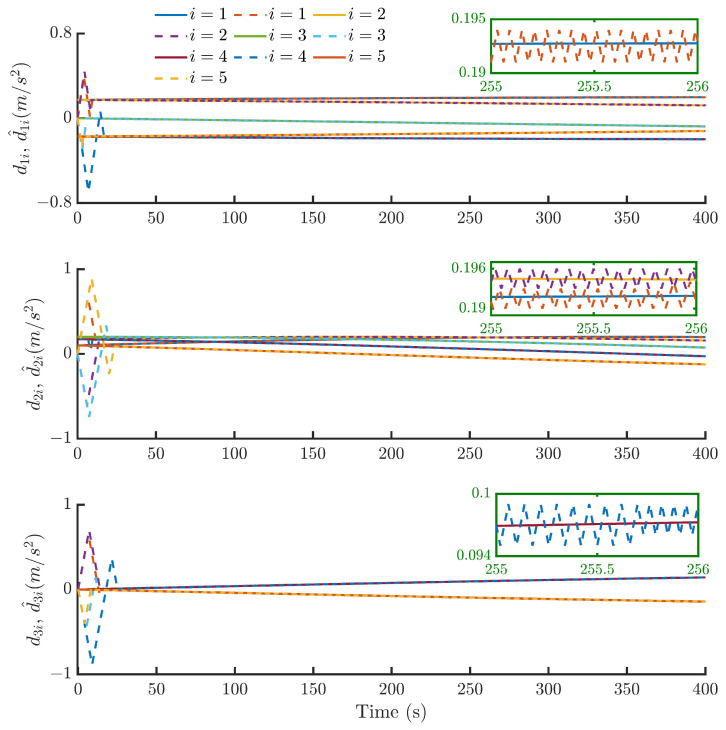
The estimates ${\hat{d}}_{i}$ (dotted lines) of the disturbances $d_{i}$ (solid lines).

**Figure 4:**
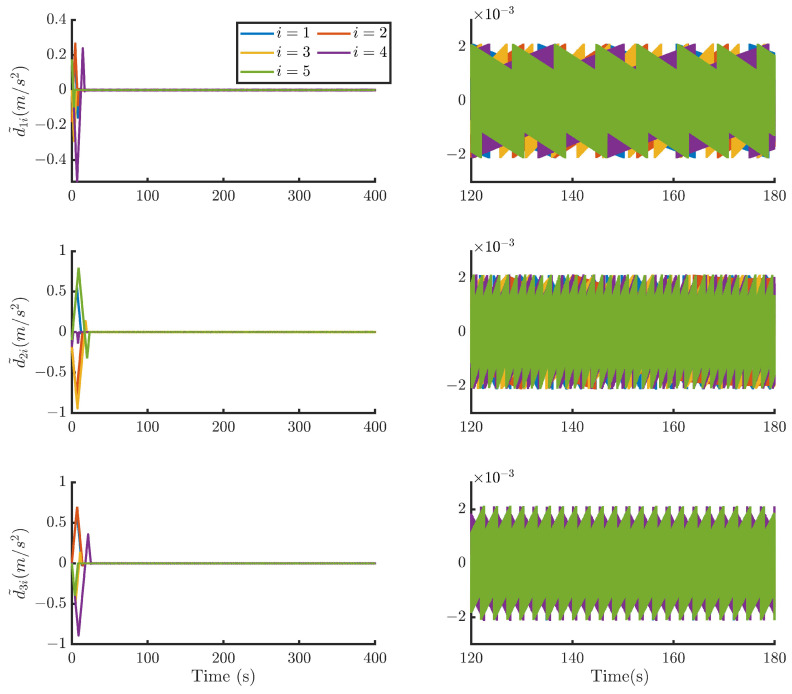
Estimation errors of $d_{i}$ of the five spacecraft.

**Figure 5:**
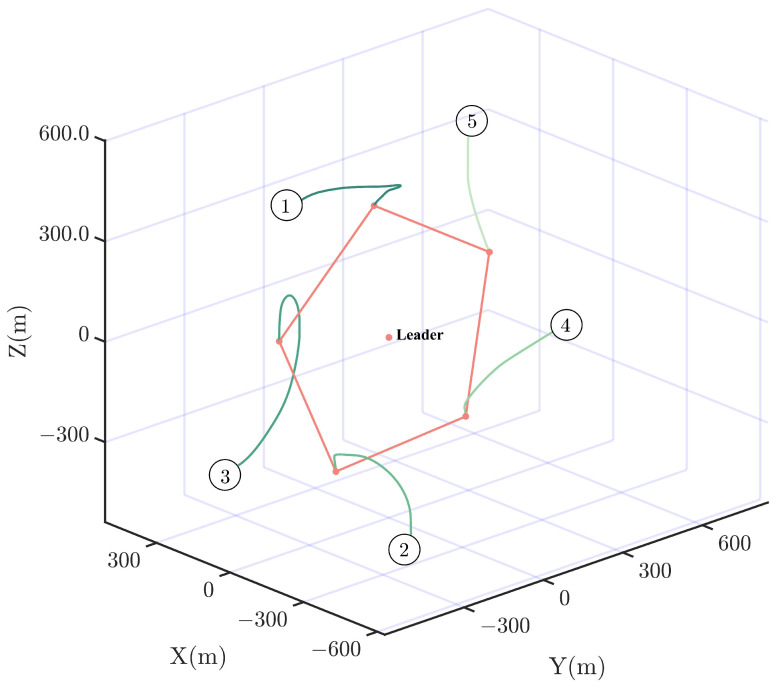
Trajectories of the spacecraft formation.

**Figure 6:**
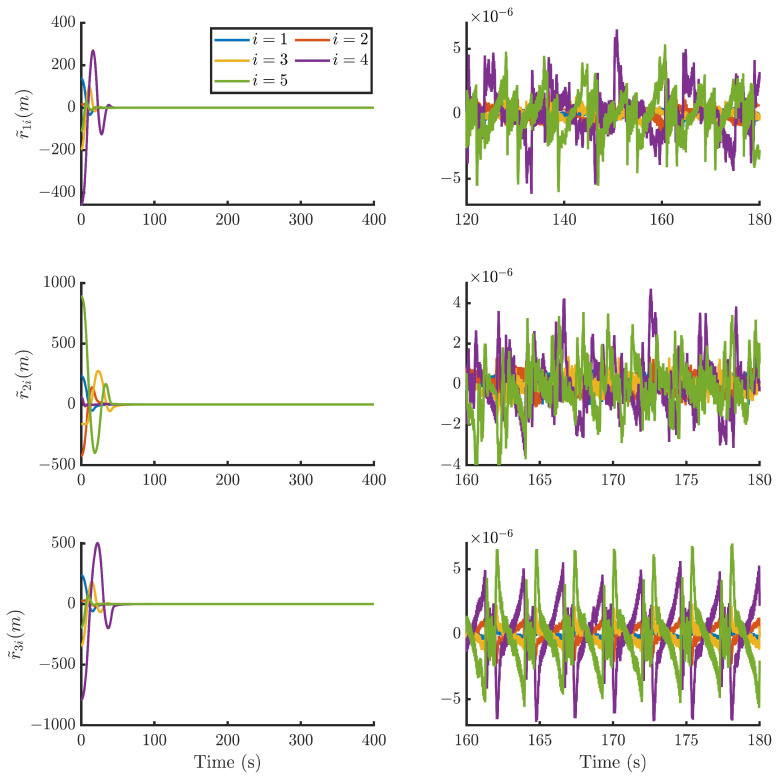
Relative position tracking errors of the five spacecraft.

**Figure 7:**
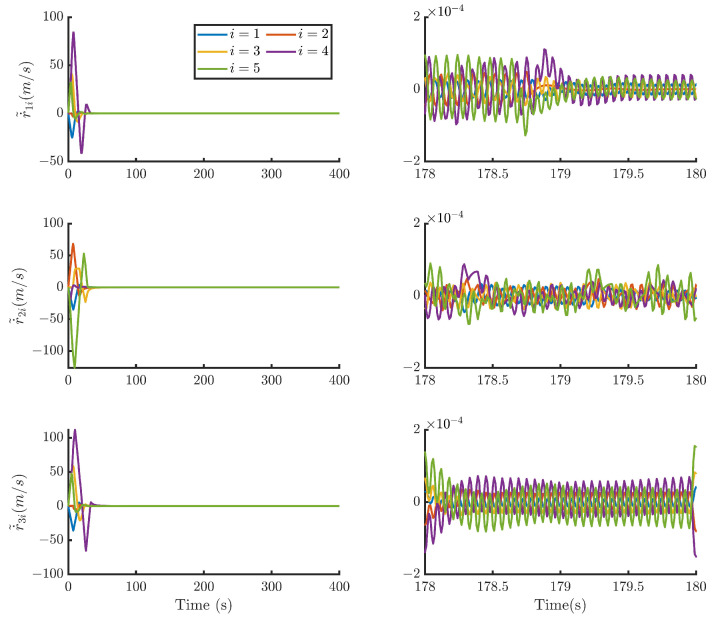
Relative velocity tracking errors of the five spacecraft.

**Figure 8:**
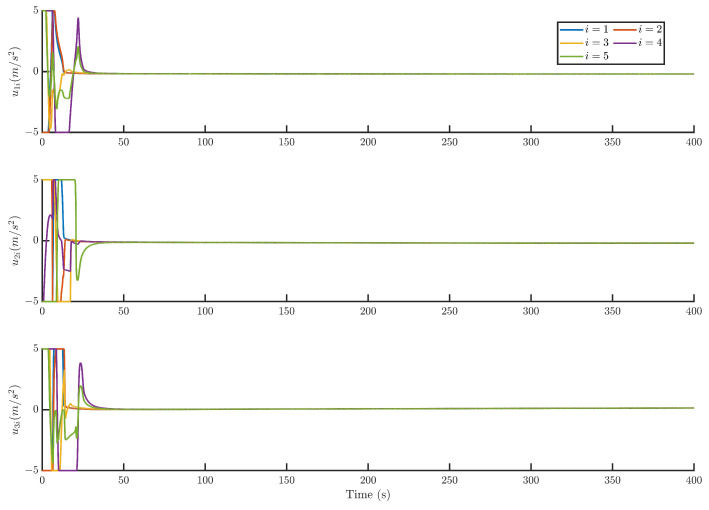
The control input of the five spacecraft.

**Figure 9:**
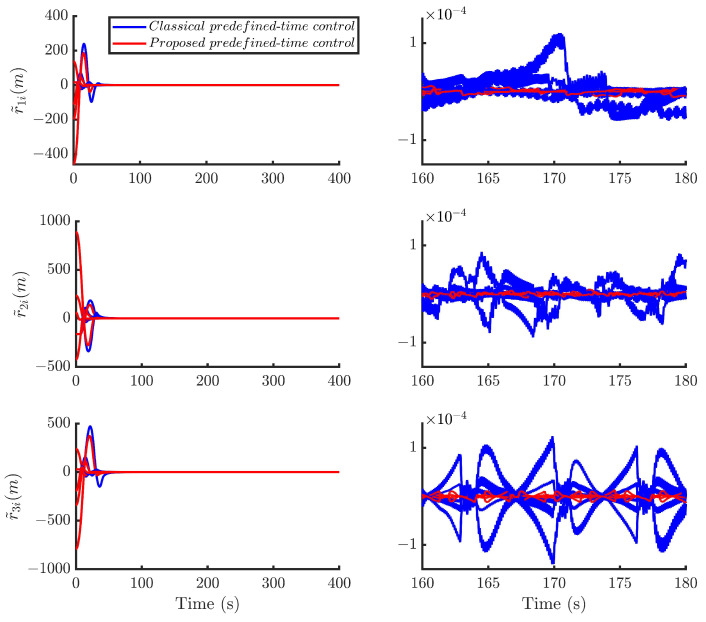
Comparison of position errors of different control methods.

**Figure 10:**
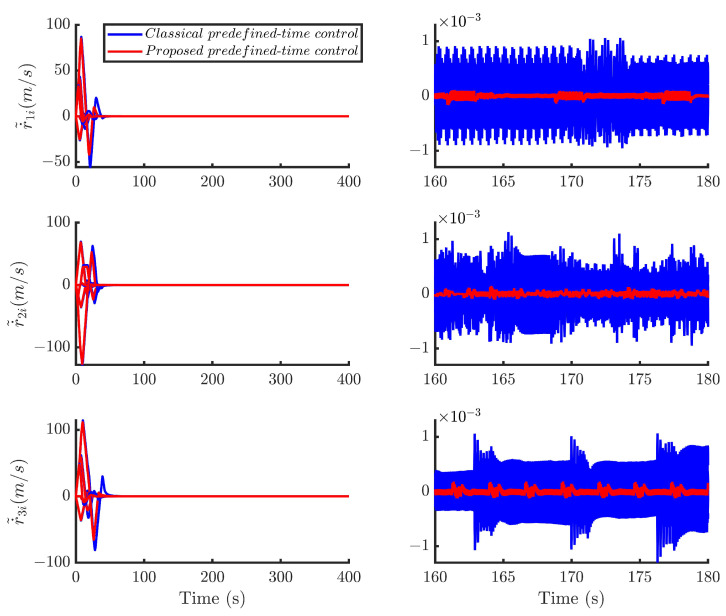
Comparison of velocity errors of different control methods.

Then, by using the control strategy, the simulation results are obtained and provided as displayed in Figure 3, Figure 4, Figure 5, Figure 6, Figure 7, Figure 8, Figure 9 and Figure 10. Figure 3 depicts the actual value and estimated value of disturbances in the spacecraft. The disturbance observer can precisely estimate the disturbances. Figure 4 shows the estimation errors of $d_{i}$. Figure 3 and Figure 4 demonstrate the estimation performance of the disturbance observer, showing that the estimation errors converge within 2 × 10^−3^ m/s^2^ and thus confirming the observer’s ability to accurately estimation time-varying disturbances. The motion of the spacecraft formation in space is depicted in Figure 5. Figure 6 and Figure 7 illustrate the tracking errors of the relative position and velocity, respectively, revealing that the desired formation is achieved within 170 s. Additionally, all the tracking errors are maintained within $7\times 10^{-6}$ m and $2\times 10^{-4}$ m/s, respectively. Figure 8 shows the control input of the five spacecraft in body-fixed frames. Notably, Figure 3, Figure 4, Figure 5, Figure 6, Figure 7 and Figure 8 depict simulations conducted under the presence of external disturbances. With the aid of a fixed-time disturbance observer that accurately estimates these disturbances, the predefined-time sliding mode control scheme proposed in this paper effectively suppresses the external disturbances, achieving rapid system convergence. This demonstrates the robustness of the proposed scheme against external disturbances. When using different initial conditions, the desired formation can also be completed in the predefined time. Similar verification results are omitted here.

Additionally, to compare different predefined-time control methods and highlight the advantages of the approach presented in this paper, we conducted a comparative analysis between the classical predefined-time control scheme and the one proposed here. According to Lemma 2, the classical control scheme employs the sliding surface and control law as shown below.

$$\begin{array}{ccc} s_{i} & = & \dot{e_{i}}+h\left(e_{i}\right),\;h\left(e_{i}\right)=\frac{\pi }{\alpha T_{P}}(V_{e_{i}}^{-\frac{\alpha }{2}}+V_{e_{i}}^{\frac{\alpha }{2}})e_{i},\\ u_{i} & = & u_{ri}-{\hat{d}}_{i}+{\left(\sum_{j=1}^{n}a_{ij}+b_{i}\right)}^{-1}\left[u_{si}+\sum_{j=1}^{n}a_{ij}\left(u_{j}+{\hat{d}}_{j}-u_{rj}\right)\right],\\ u_{ri} & = & -\left(A_{1}{\dot{r}}_{i}+A_{2}\left(r_{i}\right)\right)+{\ddot{r}}_{i}^{d},\\ u_{si} & = & -\frac{\pi }{\alpha T_{p}}(V_{s_{i}}^{-\frac{\alpha }{2}}+V_{s_{i}}^{\frac{\alpha }{2}})s_{i}-\dot{h}\left(e_{i}\right)-\delta \mathrm{sign}\left(s_{i}\right), \end{array}$$

where $V_{e_{i}}=\frac{1}{2}e_{i}^{⊤}e_{i}$, $V_{s_{i}}=\frac{1}{2}s_{i}^{⊤}s_{i}$, α∈(0,1) and $T_{p}>0$ is any given parameter.

With the identical parameters and initial conditions, Figure 9 and Figure 10 compare the position and velocity convergence errors of the two control methods, respectively. The results indicate that the predefined-time sliding mode control method adopted in this paper exhibits significant advantages in terms of error convergence precision.

## 5. Conclusions

This paper has dealt with the formation problem of MSSs by developing a predefined-time coordination control scheme. To ensure robust and high-precision control, a fixed-time disturbance observer has been designed, allowing precise estimation of these unknown disturbances within a fixed time period. By integrating the designed fixed-time disturbance observer with a predefined-time control strategy, a distributed formation control law has been proposed, which can achieve a formation of predefined-time convergence, high accuracy, and robust performance. Numerical simulations validate the effectiveness and advantages of the proposed control law. In our future work, benefiting the some excellent SMC [33,34], we will explore the predefined-time convergence challenge of MSSs with safety and reliability.

## Figures and Tables

**Table 1:** Parameters of spacecraft formation relative movement model in the simulation.

Model Parameter of Satellite Formation	Values
ω	$9.918\times 10^{-4}$ (rad/s)
$\dot{\omega }$	0 (rad/s)
$R_{l}$	6878.173 (km)
μ	3.9860047 × 10^14^ (m^3^/s^2^)

**Table 2:** The parameters of the observer and controller in the simulation.

Item	Parameters	Values
Predefined-time Controller	α	0.5
	$T_{p}$	150
	δ	diag{0.3,0.3,0.3}
Fixed-time Disturbance Observer	α	3/5
	β	7/5
	$K_{1}$	diag{2,2,2}
	$K_{2}$	diag{0.5,0.5,0.5}
	$K_{3}$	diag{0.1,0.1,0.1}

## Data Availability

The raw data supporting the conclusions of this article will be made available by the authors on request.
